# Current Knowledge on *Listeria monocytogenes* Biofilms in Food-Related Environments: Incidence, Resistance to Biocides, Ecology and Biocontrol

**DOI:** 10.3390/foods7060085

**Published:** 2018-06-05

**Authors:** Pedro Rodríguez-López, Juan José Rodríguez-Herrera, Daniel Vázquez-Sánchez, Marta López Cabo

**Affiliations:** 1 Department of Microbiology and Technology of Marine Products (MICROTEC), Instituto de Investigaciones Marinas (IIM-CSIC), 6, Eduardo Cabello, 36208 Vigo, Spain; pedrorodriguez@iim.csic.es (P.R.-L.); juanherrera@iim.csic.es (J.J.R.-H.); 2 “Luiz de Queiroz” College of Agriculture (ESALQ), University of São Paulo (USP), 11, Av. Pádua Dias, 13418-900 São Paulo, Brazil; danielvazquezsanchez@gmail.com

**Keywords:** bacteriocins, biocides, biofilm, food industry, food safety, *Listeria monocytogenes*, resistance

## Abstract

Although many efforts have been made to control *Listeria monocytogenes* in the food industry, growing pervasiveness amongst the population over the last decades has made this bacterium considered to be one of the most hazardous foodborne pathogens. Its outstanding biocide tolerance capacity and ability to promiscuously associate with other bacterial species forming multispecies communities have permitted this microorganism to survive and persist within the industrial environment. This review is designed to give the reader an overall picture of the current state-of-the-art in *L. monocytogenes* sessile communities in terms of food safety and legislation, ecological aspects and biocontrol strategies.

## 1. *Listeria monocytogenes*, a Food Safety Concern

*Listeria monocytogenes* is a ubiquitous pathogen that can stem from a febrile gastroenteritis to a severe invasive illness (listeriosis), leading to septicaemia, encephalitis, endocarditis, meningitis, abortions and stillbirths, among others syndromes [1,2]. The incidence of listeriosis is low amongst the general population, with 0.46 and 0.24 cases per 100,000 population in 2015 in the European Union and the United States respectively [3,4]. However, *L. monocytogenes* was responsible for many foodborne outbreaks with high hospitalisation and mortality rates worldwide, especially affecting pregnant women, the elderly and individuals with compromised immune systems. In particular, *L. monocytogenes* caused more foodborne outbreaks between 2005 and 2015 in the EU (83) than in the US (47), resulting in 757 and 491 cases, respectively [5,6,7,8,9,10,11,12,13]. In contrast, a higher number of cases required hospitalisation in the US (428) than in the EU (332), leading to more deceases (82 and 61 deaths respectively) and a higher mortality rate (24 and 16% of deceases related to foodborne outbreaks in the US and in the EU, respectively).

In spite of modifications to legal regulations, ready-to-eat (RTE) meats and dairy products are still the predominant vehicles involved in the main listeriosis outbreaks which have occurred since 2008, as well as other “low risk” products such as fruit and vegetables (Table 1). In addition to this, no consensus has been achieved among international food authorities in regards to the microbial criteria for *L. monocytogenes* [14]. As a matter of example, The United States Department of Agriculture (USDA) and the Food and Drug Administration (FDA) implemented a “zero tolerance” policy for *L. monocytogenes* contamination of RTE food products [15,16]. In contrast, the European Commission Regulation No. 2073/2005 (amended by EC No. 1441/2007) permits levels of *L. monocytogenes* up to 100 CFU/g in RTE foods placed on the market during their shelf-life, except in those intended for infants or for special medical purposes, in which must be absent in 25 g of product [17,18]. Canada, Australia and New Zealand also permit levels of *L. monocytogenes* lower than 100 CFU/g for RTE foods in which the growth of this pathogen is limited throughout the stated shelf-life, but it must be absent in 25 g of those which can support its growth [16,19,20]. According to the Chinese Centre for Food Safety (CFS) levels of *L. monocytogenes* of 10–100 CFU/g are allowed in RTE commercialised in China, except in those refrigerated (not frozen) in which it must be absent in 25 g of product [21,22]. In Brazil, the use of *L. monocytogenes* as microbial criteria is limited to cheese, in which it must be absent in 25 g of product [23]. Curiously, many food companies follow the national regulations for products commercialised in their own country, but not foreign regulations for products that they export, leading to products with different standards of quality and safety. These actions can involve eventual problems of cross-contamination between the processing chains and a serious risk to consumers due to this lack of universal legislation. Therefore, an international consensus in microbial criteria for foodstuffs must be reached.

In the food industry, *L. monocytogenes* can persist for months or even years on floors and equipment and in drains of food-processing facilities [35,36]. This is mainly due to its ability to survive under food-related conditions that are stressful for other bacteria, such as refrigerated temperatures, desiccation, heat and high salt content [37,38,39,40], and its ability to form biofilms [41,42]. The application of ineffective cleaning and disinfection procedures in food-processing environments, particularly in locations of difficult access, also increases the risk of establishment and growth of *L. monocytogenes* and, thus, generate continuous food product contamination [43,44]. The identification of particular niches in a food-processing facility, the validation of the efficacy of sanitation procedures applied and the continuous monitoring of the presence and reestablishment in food-processing environments are therefore required to improve the control of *L. monocytogenes*.

Livestock and produce farms are considered potential primary sources for the introduction of human pathogenic *L. monocytogenes* into the food chain and food-processing plants. In fact, *L. monocytogenes* was detected in cattle, silage, animal feeds, manure and growing grass, among others [45,46,47,48]. Nevertheless, soil, water and vegetation of natural and urban environments can also serve as reservoirs of *L. monocytogenes* [49,50,51].

*L. monocytogenes* involved in most human listeriosis cases has been isolated from RTE foods post-processed in retail facilities [52,53]. The application of inadequate post-processing procedures such as improper manipulation (e.g., bacterial transfer from operator’s hands and gloves, cutting boards or scales among others) or the use of contaminated slicing machines were the main cause of contamination in RTE foods [53,54,55,56]. In addition, *L. monocytogenes* is also found on non-food contact surfaces such as floors, drains, sinks, and walk-in cooler shelves of retail facilities [57,58]. *L. monocytogenes* can also proliferate due to temperature fluctuations in coolers during distribution and commercialisation of food products [59]. Moreover, this pathogen is detected in domestic environments [60,61,62] and public restaurants [63,64,65]. Several listeriosis outbreaks are also associated with foods purchased from or provided in hospitals and health care centres [66,67,68]. A limited knowledge of food safety, as well as an inappropriate attitude and hygiene of food handlers can directly affect the quality of the product [69,70]. Therefore, guidelines for prevention of *L. monocytogenes* contamination and persistence should be widely spread.

## 2. Efficacy of Food Industry Sanitisers against *L. monocytogenes*

According to published data, in Europe, around five trillion euros are invested annually for the implementation of hygienisation systems in food-related industrial environments. Nevertheless, the levels of bacterial contamination in processed food products is still a major issue of concern, with the increasing incidence of *L. monocytogenes* being remarkable if we take into account the notified cases of listeriosis [71]. The current tolerance to disinfectants in *L. monocytogenes* has been a topic of concern in the context of the food industry and public health regarding foodborne pathogens. The presence of high bacterial concentrations and the interference with organic matter due to insufficient cleaning prior to disinfection diminishes the activity and thus the efficacy of disinfectants commonly used in industrial premises [72]. This does not necessary mean that the quantity used is lower, but that the effective concentration of the antimicrobial is less than expected, compared to the initial amount deployed. However, anthropologic factors such as failure in dosage or inadequate rinsing are also responsible for the generation of tolerances due to the formation of areas in which sub-lethal concentrations of the disinfectant are present [73]. Additionally, it has also been stated that tolerance to certain disinfectants may contribute to the persistence of *L. monocytogenes* in the food industry [74].

In this section, the behaviour and further tolerance mechanisms to quaternary ammonium, chlorine and acid compounds in *L. monocytogenes*, are reviewed.

### 2.1. Quaternary Ammonium Compounds

Among biocides, quaternary ammonium compounds (QACs) are undoubtedly, one of the most commonly used disinfectants in the food industry efficient against bacteria, algae, fungi, spores, viruses and mycobacteria even at low concentrations [75]. QACs are active in the membrane of bacteria, casing disruption in the phospholipid bilayer and subsequent cellular content leakage causing eventual bacterial death [75]. They are stable, surface-active agents presenting low toxicity and little affected by organic matter, which make them very adequate for food industry purposes.

The described mechanisms underneath tolerances to QACs are diverse and are strongly influenced by the environment and the genetic background of each particular strain [76]. Considering the latter, a study carried out by Liu et al. [77] demonstrated how the presence of antimicrobials’ sublethal concentrations can increase the possibility of oxidative stress of the cell due to an increasing concentration of free radicals in the cytoplasm. As a result, this can promote the activation of various genetic cascades like the apparition of de novo mutations due to the triggering of the SOS-response [78]. The overuse (or misuse) of QACs, may enhance the selection of new genetic elements that can be horizontally transferred [78,79]. This fact poses an additional element for the development of new forms of tolerances in *L. monocytogenes*, thus dwindling the number of options for treatment in industrial contexts that could finally enhance the biofilm formation to this pathogen [74].

Active efflux pumps are considered so far, the main mechanism for *L. monocytogenes* tolerance to QACs. This was early described by Aase et al. [80], demonstrating an extrusion of ethidium bromide outside the cell in BAC resistant and BAC adapted strains, which not only indicated the presence of an efflux pump but also that this mechanism is intrinsic to *L. monocytogenes* and can be activated by a sublethal exposure to BAC. Subsequent studies demonstrated that these efflux pumps are chromosomically encoded and that the exposure to QACs leads to an overexpression of them [76]. Despite the general agreement on this major strategy for QAC tolerance, there is still some controversy about the origin of the genetic determinants coding for efflux pumps. As a matter of example, Dutta et al. [79] demonstrated that in BAC-tolerant *L. monocytogenes* from various sources, the *bcrABC* cassette was present in 98.6% of isolates. Contrarily, Ebner et al. [81] identified the *qacH* as the main genetic determinant in BAC resistant isolates from different food matrices, and the lack of correlation between this genotype, the isolation source, the biofilm formation capability and the serotype. More recently, a new efflux pump, *emrE*, has been described in *L. monocytogenes* conferring cross-resistance to BAC and other antimicrobials [82].

Genetic mobile elements also play an important role in the dissemination of resistance genes among *L. monocytogenes*. Among, *bcrABC*-carrying isolates, it has been proposed that the transmission and subsequent integration into the chromosome, together with other resistance genes, has been mediated via transposon-containing plasmids [79]. In addition to this, Müller et al. [83] have described in *L. monocytogenes* the structure of Tn*6188*, harbouring the *qacH* gene. Ulterior investigation regarding this mobile element, has demonstrated that cells expressing *qacH*-encoded efflux pumps, showed increased MICs to BAC and other QACs, and also a decreased susceptibility to ethidium bromide [84].

Moreover, in *L. monocytogenes*, biofilm formation itself is a cause of increased tolerance to QACs due to the alterations in the membrane fluidity of the cell [85]. These alterations are mainly because of a decrease in the proportion of iso-C15 and anteiso-C15 branched-chain fatty acids (BCFA) together with an significant increase in the quantity of saturated fatty acids (SFA) [86]. Consequently, the membrane hydrophobicity is increased, thus promoting further adherence to surfaces [87]. Similar modifications have been described in cells exposed to sublethal concentrations of BAC [73] or to cold stress [87].

### 2.2. Chlorine-Based Compounds

Chlorines are cheap and straightforwardly used antimicrobials active against bacteria, fungi and algae. Different chlorine-based compounds such as sodium hypochlorite, chlorine dioxide gas or aqueous chlorine dioxide have been proven to be active against *L. monocytogenes* [88].

Due to their fast-oxidising nature, they interact with cellular membranes or penetrate directly into the cell forming N-chloro groups that react with the cellular metabolism due to the interference with key enzymes [89]. With this regard, Valderrama et al. [90] found a *L. monocytogenes* reduction of about 4 log CFU/mL in brine chilling solutions treated with 3 mg/mL chlorine dioxide with just 90 s contact time. Nevertheless, in *L. monocytogenes*, proper chlorine efficacy seems to be age-dependent since the thickness of the cell wall in young cultures is higher, thus protecting the cells from these sanitisers. In this line, El-Kest and Marth [91] demonstrated that a solution of 1 mg/mL of free chlorine during 10 min sufficed to reduce 4.27 orders of magnitude in 48-h-old *L. monocytogenes* Scott A cultures, whereas in 24-h-old cultures the reduction was only of 2.88 orders of magnitude.

Tolerance development against chlorine-based sanitisers has been described so far to be unlikely in *L. monocytogenes* cell suspensions [92,93]. However, Lundén et al. [94] showed that continuous transfers culture in increasing concentrations of sodium hypochlorite can promote the increase in MIC values of this disinfectant. Additionally, decreased activity of chlorine-based sanitisations have been observed not because of intrinsic factors but to interactions with external elements such as organic matter [91,92,95] or divalent cations [90].

In *L. monocytogenes* biofilms, the efficacy of chlorine solutions has been proven to greatly depend on the biofilm substrate. Hence, Bremer et al. [96] observed a significant higher proportion of eliminated cells of *L. monocytogenes* when grown on stainless steel coupons compared to those grown on polyvinyl chloride surfaces. These results were also in concordance with those obtained by Pan et al. [97] demonstrating higher tolerance to chlorine treatments in those biofilms grown in Teflon compared to those on stainless steel. Moreover, it has been observed that the adaptation of planktonic cells and subsequent sessile growth on stainless steel makes biofilms to be more tolerant to chlorine, independently of the subtype, cellular density of the biofilm and its morphology [98]. Additionally, some authors also described a cross-resistance in favour of tolerance to chlorine in *L. monocytogenes* biofilms previously treated with peroxide-based products, thus indicating that the mechanisms responsible for oxidising agents’ tolerance may have a common nature in *L. monocytogenes* [97].

The effects of chlorination in *L. monocytogenes* multispecies biofilms have also been investigated. Norwood et al. [99] showed that this pathogen is able to endure concentrations higher than 1000 ppm of free chlorine in a continuous co-culture with *Staphylococcus xylosus* and *Pseudomonas fragi* on stainless steel. In contrast, other authors have found that in co-culture with *Flavobacterium* spp., a slightly acidic solution containing 400 ppm of free chlorine is enough to reduce the load both bacteria up to undetectable levels [96].

### 2.3. Acid Compounds

Acids are strong oxidisers able to interfere with cellular phospholipid bilayers and cytosolic material causing irreversible damage (e.g., disruption of proton motive force) and subsequent death to cells [100,101]. However, *L. monocytogenes* is able to adapt to low pH environments generated by natural processes (e.g., lactic fermentation) or artificially induced (e.g., acidification of water for cleaning systems) by means of different mechanisms. This not only allows this pathogen to survive in the environment, but could also increases its virulence since it further helps the bacterium to survive into the gastrointestinal tract and macrophage phagosome [102].

In spite of the fact that acid adaptation is a transient state in *L. monocytogenes* [103], it enhances the survival of this pathogen in the food industry, while also providing the bacterium with higher protection against other environmental insults [103]. Following this line, Phan-Thanh et al. [104] demonstrated that pre-exposure to mild acidic conditions (pH 5.5, 2 h) increased its endurance against lethal acidic, temperature (52 °C), salinity (25–30% NaCl) and alcoholic (15%) shocks. These effects are even more evident when the acid adaptation takes places gradually [102,105]. Additionally, it has been demonstrated that sublethal acid adaptation deeply alters the intracellular protein pattern expression, being more evident as the pH decreases [104,106], and that this differential pattern is strain-dependent [104].

There are different ways described in the literature in which *L. monocytogenes* can adapt to acidic conditions, all of them focused on the maintenance of the intracellular homeostasis. Among them, the glutamate decarboxylase (GAD) system is considered one of the major mechanisms [107]. This involves the GAD enzyme, which promotes the irreversible conversion of cytosolic glutamate to a neutral compound, the γ-aminobutyrate (GABA), by irreversible decarboxylation of the first [103]. The synthesis of GABA has a dual protective role: firstly, it consumes an intracellular proton during the process, with the subsequent increase of the pH inside of the cell [103]. Additionally, the extrusion of GABA outside the cell via a glutamate:GABA antiporter, contributes to the slight neutralisation of the pH outside the cell and the restarting of the metabolic pathway [102]. In food systems, it has been demonstrated that in glutamate-rich products, the survival rate of *L. monocytogenes* is significantly improved [107]. In addition to glutamate:GABA antiporter, other proton pumps such as F_0_F_1_-ATPase have also been proposed as active mechanisms to maintain homeostasis in acidified environments [108].

Similarly with exposure to QACs, acidic conditions modify the composition of the cytoplasmic membrane, altering the iso- and anteiso-BCFAs ratio. Giotis et al. [109] tested the response of *L. monocytogenes* 10403S to mild acid conditions, demonstrating that the total anteiso/iso ratio increased as the culture pH decreased. This was further demonstrated by Zhang et al. [110] in *L. monocytogenes* cultured in presence of various organic acids, concluding not only that the relative proportions of BCFAs were significantly altered but also that the mechanism underneath this adaptation was shared.

In biofilms, the acid-tolerance in *L. monocytogenes* seems to be strain dependent. In this line, Ibusquiza et al. [111] showed that the resistance threshold to peracetic acid between three different strains depended not only on the strain, but also the age of the biofilm and the substrate where the biofilms were grown on. These results were in accordance with those obtained by Lee et al. [112,113]. Furthermore, in addition to its overall resistance, biofilm formation is also affected when *L. monocytogenes* is exposed to acid compounds generally enhancing its adherence [114,115] even though there is evidence that early exposure to acidic conditions does not modify the ulterior biofilm formation capacity [116]. Additionally, accompanying strains, such as lactic acid bacteria, can exert a protective effect to *L. monocytogenes* in mixed-species biofilms, increasing its tolerance to acidic sanitisers [117].

## 3. Microbial Interactions and Resistance of *L. monocytogenes* Mixed-Species Biofilms

It is accepted that bacteria live in nature associated with another species forming structured multispecies biofilms [118]. Their life in communities makes unavoidable interspecies interaction and its impacts biofilm ecology.

Microbial communities can be defined as multispecies associations with complex structures that normally suppose important ecological advantages to the individual species present. In fact, it is accepted that biofilms represent a microbial phenotype with an explicit organisation level in which microorganisms are involved in intracellular interactions that can be competitive, cooperative or even neutral, depending on the microbial species involved and the environmental conditions [119].

Interspecies interactions are especially relevant in *L. monocytogenes* considering it is considered a poor biofilm former when compared to other bacterial species [120]. Several previous studies have addressed the influence of the accompanying microbiota in the number of adhered viable cells of *L. monocytogenes* in the corresponding mixed biofilm. There is a risk associated with the increased attachment of *L. monocytogenes* on food processing surfaces precolonised by other bacterial genera. In general, the number of adhered *L. monocytogenes* was increased, decreased or unaltered depending on the accompanying bacterium [121]. As an example, Norwoord and Gilmour [122] demonstrated that higher *L. monocytogenes* numbers in monocultures compared with the multispecies biofilms formed after its association with *Staphylococcus xylosus* and *Pseudomonas fragi*. Rodríguez-López et al. [123] explored the association capacity of ten different accompanying strains with *L. monocytogenes* when forming dual-species biofilms. Outcomes demonstrated a deleterious effect of several accompanying strains on *L. monocytogenes* present on biofilms in 4 out of 10 different combinations checked. On the contrary, in other studies it has been showed that accompanying strains increase the level of adherence of *L. monocytogenes* in the mixed biofilm [123,124,125,126]. In summary, literature highlights that phenomena of adhesion/aggregation between different bacterial genera cannot be predicted since different environmental conditions can be encountered within the different niches.

Generally, previously reported studies consider that the amount of adhered viable cells in biofilms is directly related with its resistance to antimicrobials [127,128]. Nevertheless, in *L. monocytogenes*, viable biomass present in the biofilm does not give any certain indication about the difficulty of this pathogen to be eliminated from a given contamination site. In fact, a study carried out by Midelet et al. [129] demonstrated that interaction of *L. monocytogenes* with *Kocuria varians* results in higher density of the first but made its detachment easier.

The specific location of *L. monocytogenes* in the mixed microbial communities seems to be crucial when thinking on the effective elimination of the cells from contaminated surfaces or foods. Sasahara and Zottola [130] described initially interactions between *Pseudomonas* sp. and *L. monocytogenes* in biofilms and claimed on the need of a primary coloniser such as *Pseudomonas* sp. for *L. monocytogenes* to attach. Curiously, subsequent confocal microscopic studies highlight that *L. monocytogenes* locates at the bottom layers of the dual biofilms with *Pseudomonas fluorescens*. Moreover, the authors argue that *L. monocytogenes* cells have to make their way towards the biofilm bottom across the matrix [131].

Specific interspecies interactions existing in nature inside the biofilm are difficult to understand because it is impossible to empirically reproduce the strategies adopted by each species of the bacterial community to finally enhance the fitness of the biofilm consortium [119]. In spite of this, several advances have been achieved, mainly referred to the role of the accompanying microbiota.

As part of biofilm fitness, resident microbiota could protect *L. monocytogenes* to external stimuli such as food processing and/or disinfection conditions. This has been a matter of concern for biofilm researchers in the last decades. However, due to the complexity associated with the experimental work within biofilms, most of the published articles had been carried out with dual-species biofilms, which can be considered an excessive simplification of the realistic situation. *Lactobacillus plantarum* protected *L. monocytogenes* from the action of BAC and peracetic acid (PAA) [117]. Similarly, Saá Ibusquiza et al. [132] also showed that the presence of *Pseudomonas putida* increased the resistance of several strains of *L. monocytogenes* to BAC and PAA. 

On the other hand, a recent study carried out by Papaioannou et al. [133] demonstrated, using a more realistic approach, that *L. monocytogenes* adhesion to stainless steel decreased (<10^2^ CFU/cm^2^) due to co-culture with indigenous microbiota commonly found in fish industry such as *Pseudomonas* spp., Enterobacteria or sulfide-producing bacteria. Furthermore, they postulated that this adhesion impairment was possibly one of the causes of an observed increased sensitivity to two common industrial disinfectants (Hypofoam and Divosan).

In spite of the difficulty associated with this type of studies, advances in microscopic and high throughput sequencing methodologies will permit to go deeper in the knowledge of species interactions in order to improve the hygienic design and the control of foodborne pathogens.

## 4. Biosanitation of *L. monocytogenes* Biofilms Using Lactic Acid Bacteria and Bacteriocins

The removal of microorganisms from food premises cannot be currently conceived without the use of conventional biocides. However, this practice has not been completely successful, and some issues have arisen. For instance, the emergence of resistant (or tolerant) strains [134,135] and a highly decreased effectiveness in the presence organic material [136] or in “harbourage sites” [121].

Huge efforts have been therefore conducted to search for new strategies of control of foodborne pathogens, with particular reference to *L. monocytogenes* [137,138,139]. This search has also included cost-effectiveness, environmentally friendly nature and low toxicity to humans as further major requirements. As a result, a number of promising alternatives have been identified, such a lactic acid bacteria (LAB), bacteriocins or bacteriophages, enzymes and surfactants (mainly as anti-adhesion and detachment agents), essential oils, electrolysed oxidising water and ozone, photocatalysts, ionising and UV radiation or ultrasonication, among others.

It is widely known that the microbiota present in food facilities can enhance or inhibit the colonisation of surfaces by *L. monocytogenes* [119,125,140]. Accordingly, Fox et al. [141] proposed to influence the microbiome in favour of antilisterial species as a strategy to reduce the presence of *L. monocytogenes*. However, the microorganisms are, in general, undesirable in food premises, since they may promote food spoilage or cause food safety problems. This is may not be the case for LAB, particularly for those having probiotic properties.

Many different LAB and several bacteriocins are known to be highly active against Gram-positive bacteria, particularly against *L. monocytogenes* [142,143]. In addition, the presence of antilisterial structural bacteriocins genes in LAB has been recently reported [144]. Accordingly, several studies have examined the potential of LAB and their bacteriocins as a tool for the control of biofilms of *L. monocytogenes* in food facilities. This last section of this review is intended to briefly outline some of the most significant results of these studies.

### 4.1. Preventing Biofilm Formation

Nisin is, without any doubt, the most studied bacteriocin. Moreover, among bacteriocins, only nisin has been granted a generally recognised as safe (GRAS) status by the FDA and approved for use as a food additive (additive number E234) within the European Union. Initial studies were, therefore, focused on the effectiveness of nisin and nisin-producing *Lactococcus lactis* subsp. *lactis* strains against biofilms. An early study by Bower et al. [145] already showed that nisin films adsorbed on silica surfaces inhibited the growth of *L. monocytogenes*. In addition, high nisin concentrations were found to be lethal to attached cells.

Since then, a number of studies have evaluated the potential of several bacteriocins to prevent adhesion and biofilm formation of *L. monocytogenes* on different plastic and metallic substrates, specifically nisin [146,147,148], enterocins [148,149] and sakacin 1 [150]. Although these studies were conducted using from pure bacteriocins to cell-free extracts, results clearly show that bacteriocins may delay but not prevent biofilm formation completely. In fact, only enterocin AS-48 (conditioned on polystyrene surfaces) was reported to be able to completely inhibit biofilm formation for at least 24 h, but longer times of study were not tested [149]. Similarly, Winkelströter et al. [150] also observed a noticeable inhibition of initial stages of biofilm formation for up to 24 h in the presence of a cell-free neutralised supernatant of *Lactobacillus sakei* 1 (containing non-purified sakacin 1), but regrowth of biofilms took place subsequently, which was attributed to a possible lack of competition for nutrients or a selection of bacteriocin-tolerant phenotypes.

The effect of *Lactococcus lactis* CNRZ 150, a nisin-producing strain, against the attachment of *L. monocytogenes* was also examined [151]. These authors underlined an additional advantage of using bacteriocin-producing LAB over bacteriocins, that is, competitive inhibition limiting nutrient supply and, accordingly, two different approaches were addressed. The first, “deferred adhesion” (later defined as exclusion mechanism), consists of testing the effect of pre-formed *L. lactis* biofilms. In the second, “simultaneous adhesion” (later, competition mechanism), the effect is tested by co-culturing *L. lactis* and *L. monocytogenes*. In both scenarios, attachment of *L. monocytogenes* and subsequent biofilm formation was effectively prevented.

Considering that it is highly likely that *L. monocytogenes* encounters resident biofilms rather than solid abiotic surfaces in food-processing environments [152], the effectiveness of LAB against the attachment of *L. monocytogenes* has been interchangeably tested in terms of exclusion or competition mechanisms in many subsequent studies. This effect has been defined as competitive exclusion. As a result, a high number of different strains has been shown to be highly effective: *Enterococcus durans* [153,154], *Enterococcus faecium* [146], *L. lactis* [153,154,155], *Lactobacillus plantarum* and *Enterococcus casseliflavus* [156], *Leuconostoc mesenteroides* [157], *L. sakei* [150,158], *Pediococcus acidilactici*, *Lactobacillus amylovorus* and *Lactobacillus animalis* [159], *Lactobacillus acidophilus*, *Lactobacillus casei*, *Lactobacillus paracasei* and *Lactobacillus rhamnosus* [160], and *Lactobacillus paraplantarum* [161], among others.

Generally, bacteriocin-producing strains have been found to be more effective than non-bacteriocin-producing strains against biofilm formation by *L. monocytogenes*. This was clearly found for *E. faecium* [146], *L. mesenteroides* [157] and *L. sakei* [150]. However, the effectiveness of LAB can be also due to other antimicrobial metabolites, such as lactic acid and other organic acids which also decrease pH, as well as biosurfactants that can additionally prevent adhesion [155,162].

Additionally, competition for adhesion sites and nutrients was also shown to inhibit biofilm formation [163,164]. Interestingly, a study performed by Habimana et al. [164] showed by confocal laser-scanning microscopy of dual-species biofilms formed by co-culture with *L. lactis* that *L. monocytogenes* cells were located in the bottom layers of biofilms, entirely covered by *L. lactis*. In addition, modelling revealed that *L. monocytogenes* would be, in their own words, smothered by competitors and forced into a survival lifestyle, rather than into proliferation or colonisation processes. This inhibition would mainly occur during the initial phases of biofilm formation, essentially due to longer generation time and latency of *L. monocytogenes*. A similar effect had been already detected by Leriche et al. [151], who found that *L. monocytogenes* became permanent resident in dual-species biofilms when the inoculum size was high (10^8^ CFU/mL), even though high densities of *L. lactis* were able to outcompete and prevent *L. monocytogenes* to resume growth on surfaces. In keeping with these studies, it is worth to indicate that *L. monocytogenes* was found to be more resistant to disinfection in dual-species biofilms with *L. plantarum* than in single-species biofilms, particularly when outnumbered by *L. plantarum*, which seems to indicate a protective effect of the latter [117].

### 4.2. Removal of Biofilms

In line with these above mentioned studies, different comparative studies have shown that the effectiveness of LAB against pre-formed biofilms of *L. monocytogenes* (approach known as displacement mechanism) is significantly lower than on adhesion and biofilm formation by competitive exclusion [158,159,160]. That is, acting early would seem to be most appropriate to prevent biofilm formation. A similar conclusion can be drawn by comparing results from two different studies conducted by Zhao et al. [153,154], despite the effectiveness of displacement being considerably increased by extending treatments with LAB for up to 3 weeks.

Similarly, nisin was found to act rather slowly and, more importantly, with a limited effectiveness against pre-formed biofilms of *L. monocytogenes* [111,155]. This was attributed to a reduced ability to diffuse into the matrix and reach cells. Subsequent studies have confirmed that nisin does not seem to be practical as a surface sanitiser on its own [165,166]. Biofilms were also highly resistant to treatments with enterocin [149] and a semi-purified curvacin extract of *L. sakei* [158]. Concentrated cell-free supernatants from several bacteriocin-producing LAB did not have strong effects on pre-formed biofilms of *L. monocytogenes* either [167]. On the contrary, an important effect on biofilms was recently claimed for both nisin and enterocin [148], but rather high cell densities could be still clearly observed by scanning electron microscopy following treatments.

### 4.3. Combined Treatments

*L. monocytogenes* can develop tolerance and even resistance to bacteriocins if exposed to sub-inhibitory concentrations [168,169], and this decreases substantially the efficacy of treatments. Thus, Bower et al. [145] had already shown that coating surfaces with nisin did not inhibit the adhesion of nisin-resistant *L. lactis*. Combining LAB or bacteriocins with other antimicrobial factors may provide a greater effect, something that has been widely known for a long time. Thus, Leriche et al. [151] had already suggested the use of hurdle technology-like approaches to overcome bacteriocin resistance. Some studies have tested this strategy on biofilms of *L. monocytogenes*.

Of note, the treatment of floor drains of food-processing facilities with one strain of *L. lactis* subsp. *lactis* and other of *E. durans* greatly reduced the contamination with *L. monocytogenes* [154,170]. This combination should reduce the likelihood that *L. monocytogenes* developed tolerance to nisin too. Thus, most drains were found to remain free of detectable *L. monocytogenes* for several weeks after completing treatments.

Remarkably, bioencapsulation of thermally-treated fermentates of two strains of *Carnobacterium maltaromaticum* and one of *Enterococcus mundtii*, plus a relatively high nisin concentration, in an alginate matrix supported by a mesh-type fabric was highly effective against biofilms of *L. monocytogenes* [171]. Bioencapsulation allows bacteriocins to be slowly released, which seems to be more effective than large doses [172], as long as the emergence of resistance does not occur. This biocontroller eliminated *L monocytogenes* from biofilms formed in floor gutters in a fish processing plant after only 48 h of contact time, but was rather ineffective against biofilms formed on plastic surfaces (i.e., Teflon and rubber), where they were thinner and the attachment was stronger than on stainless steel. Importantly, conventional biocides did not reduce the effectiveness of the biocontroller. They were thus used jointly to achieve maximum effectiveness.

As an alternative to combine different LAB, some researchers have proposed to combine bacteriocins with different modes of action. This involved merging nisin—a class I bacteriocin—with enterocin—belonging to class IIb—, a bacteriocin produced by enterococci, was highly active against biofilms of nisin-resistant *L. monocytogenes*. Four-fold less of both bacteriocins were required and, importantly, no cross-resistance was detected [148]. On the contrary, cross-resistance for nisin and class IIa bacteriocins has been detected [173,174]. Nonetheless, previous studies demonstrated that some enterocins can present cytotoxicity upon epithelial cells [175], hence, whether they can be safe for use in food environments still remains to be clarified.

Combining bacteriocins with conventional biocides also seems an attractive strategy to reduce the likelihood of colonisation by resistant variants. Thus, the combination of enterocin AS-48 with different commercial sanitisers (quaternary ammonium compounds, bis-phenols or guanides) was found to be much more effective than any single treatment, but this effect was not observed with oxidising agents [149]. This approach would also allow conventional biocides to be used in lower amounts while increasing efficacy, which is highly important to reduce toxicity to humans and in the environment.

### 4.4. Final Considerations

A controlled application of LAB seems a very promising approach to prevent or even remove *L. monocytogenes* from food facilities basically as a result of a high competitive potential for adhesion sites and nutrients, and the production of some growth-inhibiting compounds, majorly bacteriocins. Moreover, the ability of LAB to spread and colonise surfaces can make them highly suitable as an alternative treatment for difficult-to-reach locations, where *L. monocytogenes* is not easily removed by routine cleaning and disinfection.

LAB have been safely used by humans for centuries in food production and preservation. However, they have no legal status for use as biosanitisers in the food industry. Accordingly, some issues have arisen concerning the use of some LAB. Prerequisites for a safe use need to be clearly defined.

Ideally, bacteriocin-producing LAB with no cross-resistance should be strategically combined to increase efficacy and prevent the emergence of bacteriocin-resistant phenotypes. In this sense, studies intended to validate different combinations of LAB should be encouraged. However, LAB generally join pre-existing polymicrobial biofilms in food processing environments rather than forming new structures. Consequently, this coexisting microbiota as well as temperature (which fluctuates rather significantly), the surface or soiling, among other factors, can eventually affect attachment, growth and bacteriocin production of each LAB, and therefore the effectiveness of treatments. Treatments should be therefore optimised individually. Unfortunately, only a small number of studies have addressed in situ testing [154,170,171], which makes it highly likely that applications are far from being straightforward. The design of strategies for in situ application of LAB in the food industry is thus needed.

## 5. Conclusions and Future Perspectives

There is no doubt that the recalcitrance of *L. monocytogenes* in foodstuffs is greatly influenced by the ubiquitous presence of its biofilm among food contact and non-food contact surfaces within food-processing premises [121]. Despite the great advances that have been made over the last few decades in the field of food safety, several outbreaks (Table 1) with high rates of morbidity and mortality, especially within the so-called YOPI (young, old, pregnant, immunosupressed) group, are still reported [71,176].

Understanding the various genetic and physiological underlying mechanisms leading to antimicrobial resistance as well as the influence on *L. monocytogenes* of pre-existing resident/transient microbiota and vice versa, are nowadays considered as key factors to developing fast, efficient, safe and cost-effective treatments in order to improve the environmental control of this foodborne pathogen.

Additionally to biocontrol as presented in this review, there is a significant amount of ongoing investigation developed by several groups focused on the design of ad hoc antibiofilm strategies such as enzymes [177], bacteriophages [178] or combined strategies [179]. Nevertheless, the rapid adaptation undergone by the different members of sessile communities makes us always being one step behind. Hence, the development of preventive rather than disinfecting strategies based on case-by-case approaches appears as wide field of research to go in-depth to eventually ensure the quality and safety of foodstuffs consumed in the society.

## Figures and Tables

**Table 1:** Main outbreaks of foodborne listeriosis since 2008.

Year	Country	Food Product	Cases	Hospitalisations	Deaths	Ref.
2008	Canada	Delicatessen meat	57	47	24	[24]
2009–2010	Austria, Germany and Czech Republic	“Quargel” cheese	34	34	8	[25]
2009–2012	Portugal	Fresh cheeses	30	30	11	[26]
2010	Texas (US)	Diced celery	10	10	5	[27]
2011–2012	28 US states	Cantaloupes	147	143	33	[28]
2012	14 US states	Brand ricotta salata cheese	22	20	4	[29]
2012	Spain	Latin-style fresh cheese	2	2	2	[30]
2013–2014	Switzerland	RTE salad	32	32	4	[31]
2013–2014	Denmark	RTE meat products	41	41	17	[32]
2014–2015	12 US states	Caramel apples	35	34	7	[33]
2015	10 US states	Soft cheeses	30	28	3	[34]
2016	9 US states	Packaged salads	19	19	1	[3]
2016	4 US states	Frozen vegetables	9	9	3	[13]

RTE: ready-to-eat.
